# *Dirofilaria immitis* antigenemia and microfilaremia in Iberian wolves and red foxes from Portugal

**DOI:** 10.1186/s13071-022-05170-5

**Published:** 2022-05-09

**Authors:** Sónia Gomes-de-Sá, Sérgio Santos-Silva, Alícia de Sousa Moreira, Patrícia Ferreira Barradas, Irina Amorim, Luís Cardoso, João R. Mesquita

**Affiliations:** 1 ICBAS–School of Medicine and Biomedical Sciences, Porto University, Porto, Portugal; 2 Epidemiology Research Unit (EPIUnit), Instituto de Saúde Pública da Universidade Do Porto, Porto, Portugal; 3 Institute of Molecular Pathology and Immunology of the University of Porto (IPATIMUP), Porto, Portugal; 4 Institute for Research and Innovation in Health (i3S), University of Porto, Porto, Portugal; 5 Department of Veterinary Sciences and Animal and Veterinary Research Centre (CECAV), University of Trás-os-Montes e Alto Douro (UTAD), Vila Real, Portugal

**Keywords:** *Dirofilaria immitis*, Foxes, Wolves, Portugal, Wildlife

## Abstract

**Background:**

*Dirofilaria immitis* is a parasitic nematode endemic in the Mediterranean countries, which causes cardiopulmonary dirofilariosis in wild and domestic animals. Despite being recognized hosts of *D. immitis*, wild carnivores such as wolves and foxes are frequently disregarded when considering a potential role in the transmission of these zoonotic nematodes. In Portugal, studies available regarding *D. immitis* circulation are scarce, likely underestimating its relevance. To add knowledge on this, we sought to assess Iberian wolves (*Canis lupus signatus*) and red foxes (*Vulpes vulpes*) from northern Portugal for *D. immitis* antigenemia and microfilaremia.

**Methods:**

Blood samples from 42 Iberian wolves and 19 red foxes were collected, during 2010–2012, in Peneda-Gerês National Park. Antigenemia was searched for by rapid antigen detection test kits (Uranotest Dirofilaria ®). Microfilaremia was assessed by polymerase chain reaction (PCR). Nucleic acids were extracted from blood using QIAamp® DNA Mini Kit (Qiagen), and DNA was screened for the presence of microfilaria using a conventional PCR targeting the 5.8S-internal transcribed spacer 2–28S regions, followed by bidirectional sequencing, Basic Local Alignment Search Tool analysis and phylogenetic analysis.

**Results:**

Three red foxes had antigenemia, with an occurrence of 15.8% (95% confidence interval [CI] 3.4–39.6), while showing no evidence for the presence of microfilaremia. No wolf samples presented evidence for *D. immitis* antigenemia. Nevertheless, two wolves were positive for *D. immitis* microfilaremia (4.8%; 95% CI 0.6–16.2%) as revealed by PCR and confirmed by bidirectional sequencing.

**Conclusions:**

Although *Dirofilaria* microfilaremia in wolves does not necessarily correlate to an endangerment of the infected animal's health, positive individuals can act as a reservoir for further infection if the intermediate mosquito hosts are present. To the best of our knowledge, one single study had reported that wolves were suitable *Dirofilaria* hosts, but microfilaremia have never been reported.

**Graphical Abstract:**

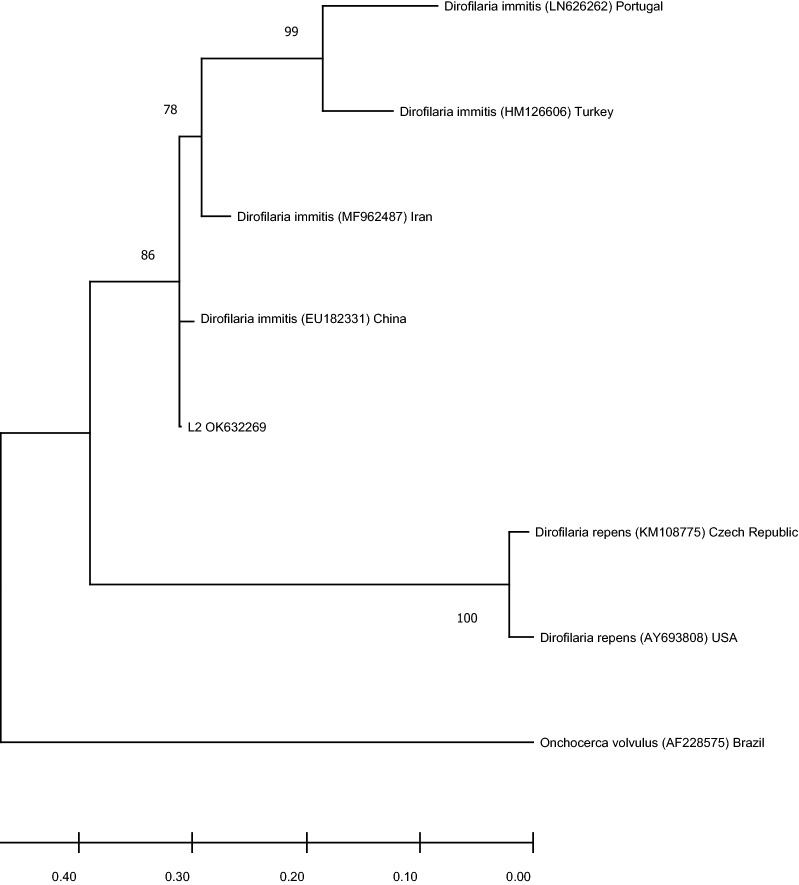

## Background

*Dirofilaria immitis* (Rhabditida; Onchocercidae) is a parasitic nematode, which causes cardiopulmonary dirofilariasis in wild and domestic canines and felines, and also pulmonary dirofilariasis in humans [1]. Mosquitoes (Diptera; Culicidae) are vectors to *Dirofilaria* spp., making the parasite distribution susceptible to changes, as well as to rapid and significant variations in defined geographic regions, such as movement of infected animals, the introduction of new mosquito species and anthropogenic activities in ecosystems [2, 3].

Dirofilariosis is endemic in the Mediterranean countries, including Portugal, particularly because of the appropriate geographic and climatic conditions [4]. The climate in Portugal is typically temperate with warm and dry summers. It is considered to be divided in subtypes, namely the first with hot summers and average temperatures > 22 °C in the warmest months and the second with warm summers with average temperatures ≤ 22 °C in the warmest months and with ≥ 4 months with the average temperatures > 10 °C [5].

Wildlife carnivores have long been overlooked when considering their possible role in transmitting zoonotic nematodes [6]. However, increasing human activities have continuously promoted wild environment invasion, ultimately redefining domestic and wild interface boundaries and consequently increasing the contact between humans and wild animals [6]. Moreover, wild animals are frequently exposed to vector-borne pathogens to such an extent that wild carnivores like the gray wolf (*Canis lupus*), red fox (*Vulpes vulpes*) and golden jackal (*Canis aureus*) are now recognized hosts of *D. immitis* [7].

Wolves and foxes’ role as *D. immitis* reservoir hosts and their contribution to disease transmission are acknowledged throughout Europe [8]. However, in Portugal, little information is known regarding *D. immitis* circulation, and the studies available are limited to a few surveys and case reports, possibly underestimating the relevance of these nematodes [3]. Moreover, the extent to which wild carnivores remain reservoirs for *D. immitis* is still unknown. As such, the aim of the present investigation was to assess the presence of *D. immitis* in wolves and foxes in Portugal by searching for both *D. immitis* antigens, using a rapid immunomigration test (RIM), and for *D. immitis* microfilaremia, by using a species-specific polymerase chain reaction (PCR) assay optimized for the simultaneous detection and differentiation of *D. immitis* and eight other concurrent filarioids.

## Methods

Blood samples were collected from a total of 61 wild carnivores, 42 wolves (*C. lupus signatus*) and 19 foxes (*V. vulpes*), during 2010–2012, from a National Park located at the northern border of Portugal (Peneda-Gerês National Park). Samples from these animals were collected as part of a carnivore protection program, an initiative that manages the collection of recently deceased animals suspected to be poisoned. Tissues and other matrices are available for studies considered to be of value for the assessment of animal population morbidities and mortalities.

For the initial detection of *D. immitis* infection, rapid antigen detection test kits (Uranotest Dirofilaria ®, Barcelona, Spain) were used, according to the manufacturer’s instructions. This test consists of an immunochromatographic technique aiming at the qualitative detection of the *D. immitis* antigen in blood, which resources to 14 kDa antigen detection not related to the parasite’s feminine genital apparatus, hence detecting both male and female parasites. The platform detects infections with a load of only one adult parasite of any type (males, adult females, immature females), with sensitivity and specificity of 94% and 100%, respectively, compared to necropsy, according to the manufacturer’s information.

To detect microfilaremia, DNA was extracted from blood using the QIAamp® DNA Mini Kit (Qiagen, Valencia, CA, USA), using an automated QIAcube nucleic acid extractor (Qiagen GmbH, Hilden, Germany). DNA was stored at − 20 °C until further analysis. A negative extraction control was processed along with each batch of 12 samples. DNA specimens were initially screened for the presence of microfilaria by using a conventional PCR targeting the 5.8S-internal transcribed spacer (ITS) 2–28S regions of the genome. Briefly, pan-filarial primer pair − DIDR-F1 and DIDR-R1 − amplifying products with distinct molecular weights were used to differentiate nine filarial species, namely *Acanthocheilonema dracunculoides* (584 base pairs [bp]), *Acanthocheilonema reconditum* (578 bp), *Brugia malayi* (615 bp), *Brugia pahangi* (664 bp), *Brugia timori* (625 bp), *Dirofilaria immitis* (542 bp), *Dirofilaria repens* (484 bp), *Mansonella ozzardi* (430 bp) and *Onchocerca volvulus* (470 bp). For all reactions, a total of 5 μl of genomic DNA was added to 12.5 μl Xpert Fast Hotstart Mastermix (2×) with dye (GRiSP, Porto, Portugal), 5.5 μl of deionized sterile water and 1 μl (10 μM) of each of the DIDR-F1 and DIDR-R1 primers in a 25-μl final volume of the reaction mixture. The reactions were carried out in an automatic DNA thermal cycler 100 (Bio-Rad Laboratories, Hercules, CA, USA), including negative and positive controls (extracted from an adult female *D. immitis*). The PCR amplification products were visualized by Xpert Green DNA Stain direct (GRiSP, Porto, Portugal) fluorescence after electrophoresis in a 1.5% agarose gel at 100 V for 40 min. To confirm species identification, all amplicons of expected size were sequenced bidirectionally for genetic characterization. Briefly, amplicons were purified with GRS PCR & Gel Band Purification Kit (GRiSP, Porto, Portugal), and bidirectional sequencing was performed by Sanger method, using the respective primers. Sequences were manually corrected using the BioEdit Sequence Alignment Editor v 7.1.9 software package, version 2.1 (Ibis Biosciences, Carlsbad, CA, USA), and further analyses were performed by comparison with the sequences available in the NCBI (GenBank) nucleotide database (http://blast.ncbi.nlm.nih.gov/Blast). Phylogenetic analysis was performed using MEGA version 6.0 software [9]. The obtained consensus sequences in this study and representative sequences for *O. volvulus*, *D. immitis* and *D. repens* obtained from GenBank were used for the phylogenetic analysis. Maximum likelihood (ML) method was applied. The ML bootstrap values were estimated using 1000 replicates with Tamura 3‐parameter as the correction model, estimated as the best substitution model by MEGA version 6.0 software. We deposited the 5.8S-ITS2-28S of *D. immitis* sequences recovered in this study in GenBank.

## Results and discussion

**Fig. 1:**
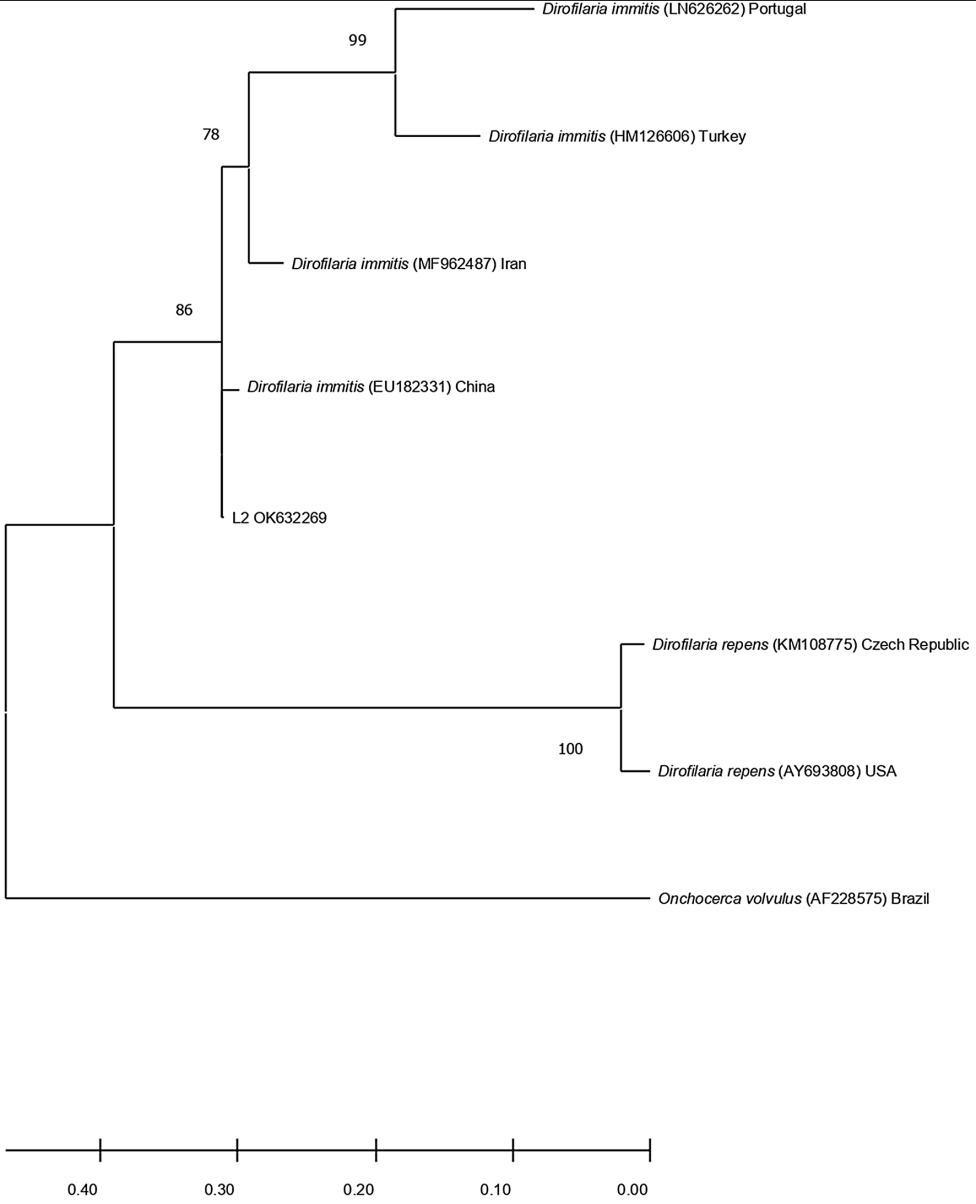
Phylogenetic analysis of *D. immitis* found in wolves in Portugal. The evolutionary history was inferred by using the ML method based on the Tamura 3‐parameter model. The analysis involved eight sequences. The *D. immitis* sequence characterized in this study is represented by sample code (L2) followed by the GenBank accession number (OK632269). Other sequences are represented with the species name followed by the corresponding accession number and country. Branch lengths are measured as the number of substitutions per site. Reliability of internal branches was assessed using the bootstrapping method (1000 replicates)

For the initial detection of *D. immitis* infection, out of the 61 wild carnivores screened by rapid antigen detection, three had *D. immitis* antigen circulation in blood (4.9%; 95% confidence interval [CI] 1.0 − 13.7). All three wild carnivores showing antigenemia were foxes, yielding an occurrence of 15.8% (95% CI 3.4−39.6). The same 61 animals were again tested for microfilaremia through conventional PCR, and only two wolves (1.6%; 95% CI 0.0−8.8) tested positive, with a prevalence in the whole sampled lupine population of 4.8% (95% CI 0.6 − 16.2). Both positive samples yielded amplicons with 542 bp, being presumptively positive for *D. immitis.* After bidirectional sequencing, the consensus sequences showed to be identical. Basic Local Alignment Search Tool confirmed the identity (100%) of *D. immitis* in both lupine samples. The following accession numbers were assigned to the sequences obtained in this work: OK632269 and OK632270. One of the sequences obtained (OK632270) spanned only 77 nucleotides (nt); hence, phylogenetic analysis was performed with the other sequence. Phylogenetic analysis based on the 460 nt partial region of the 5.8S-ITS2-28S regions showed clustering with *D. immitis* (Fig. 1).

The present study evaluated *D. immitis* occurrence in a population of Portuguese wolves and foxes by searching for both *D. immitis* antigens and *D. immitis* microfilaremia, using a RIM test and a species-specific PCR assay followed by sequence confirmation, respectively. *Dirofilaria immitis* is usually detected by specific antigen testing and/or identification of microfilariae [1]. Despite the negative heartworm antigen test result, the infected animals may still show microfilaremia in the blood [1]. In Europe, eight species of filarioids, including zoonotic species, have been reported mainly in domestic dogs, and occasionally in wild carnivores [10]. Species discrimination is of high clinical and epidemiological importance because of zoonotic concerns and therapeutic implications [1]. The application of molecular analysis targeting filarial DNA is a highly sensitive and specific analytical tool for the diagnosis and simultaneous characterization of filarial infections, thus being an extremely valuable approach [1].

In this study, none of the 42 wolf samples presented evidence for *D. immitis* antigenemia. Nevertheless, two wolves were positive for *D. immitis* microfilaremia by PCR (4.8%; 95% CI 0.6–16.2%). Although *Dirofilaria* microfilaremia does not necessarily correlate to an endangerment of the infected animal's health, the individual can act as a reservoir for further infection if the intermediate mosquito host is present [1]. To the best of our knowledge, only one study has reported that wolves were suitable *Dirofilaria* hosts and appeared exposed to infection similarly to sympatric unprotected dogs [7]. Until today, microfilaremia had never been found, thus hampering the assessment of the impact of wolves on infection maintenance.

Interestingly, the present study shows that none of the foxes presented microfilaremia, but three were positive for *D. immitis* antigenemia (15.8%; 95% CI 3.4–39.6). Variable occurrences have been reported, namely 6.4% in Australia [11], 32.3% in Spain [12], 1.6% in Serbia [13], 3.7% in Hungary [14], 25.2% in Bulgaria [15], 0.3% in Romania [10] and 2% in France [16]. In Portugal, the prevalence of *D. immitis* detected by necropsy of red foxes ranged from 3.2% in northern-central locations, such as Coimbra district [17], to 11.8% in southern and central districts, such as Santarém and Setúbal [18]. In a serological survey conducted in red foxes in Portugal, 8.5% (10/118) were positive for *D. immitis* circulating antigens, with positive animals found in northern and southern areas [3]. It should be noted that the lack of microfilaremia can be related to several factors, including unisexual infections, pre-patency or the host's immune response leading to the elimination of microfilariae. Comparisons between studies should be made with caution as different assays with distinct sensitivities/specificities were used.

## Conclusions

The present study provides molecular and serological evidence for *D. immitis* infection in wild carnivore species present in Portugal, supporting their potential epidemiological role. While in endemic areas frequent chemoprophylactic treatments of domestic dogs reduce the overall prevalence of the infection, wild canids might play a crucial role in the maintenance of infection. Understanding infection and disease prevalence in wild canids is especially important because these may act as reservoirs, increasing the risk of infection for domestic pets, including urban canids [19]. Infected microfilaremic carnivores may, in the presence of competent vector species, also act as reservoir hosts. To the best of our knowledge, this is the first detection of *D. immitis* microfilaremia in wolves, supporting that these animals can have a role as *D. immitis* reservoirs.

## Data Availability

Data supporting the conclusions of this article are included within the article.

## Abbreviations

bp: Base pairs
CI: Confidence interval
ITS: Internal transcribed spacer
ML: Maximum likelihood
nt: Nucleotide(s)
PCR: Polymerase chain reaction
RIM: Rapid immunomigration
